# Herb-anticancer drug interactions in real life based on VigiBase, the WHO global database

**DOI:** 10.1038/s41598-022-17704-z

**Published:** 2022-08-19

**Authors:** Stéphanie Pochet, Anne-Sophie Lechon, Cécile Lescrainier, Carine De Vriese, Véronique Mathieu, Jamila Hamdani, Florence Souard

**Affiliations:** 1 Department of Pharmacotherapy and Pharmaceutics, Faculty of Pharmacy, Université Libre de Bruxelles (ULB), Boulevard du Triomphe, CP 205/07, Access 2, Campus de la Plaine, Building BC, 1050 Brussels, Belgium; 2 Vigilance Division, Cell Human Pharmacovigilance Evaluation, Federal Agency for Medicines and Health Products (FAMHP), Brussels, Belgium

**Keywords:** Cancer therapy, Pharmacology, Pharmacodynamics, Pharmacokinetics, Secondary metabolism

## Abstract

Cancer patients could combine herbal treatments with their chemotherapy. We consulted VigiBase, a WHO database of individual case safety reports (ICSRs) which archives reports of suspected Adverse Drug Reactions (ADRs) when herbal products are used in conjunction with anti-cancer treatment. We focused on the possible interactions between antineoplastic (L01 ATC class) or hormone antagonists (L02B ATC class) with 10 commonly used herbs (pineapple, green tea, cannabis, black cohosh, turmeric, echinacea, St John’s wort, milk thistle and ginger) to compare ADRs described in ICSRs with the literature. A total of 1057 ICSRs were extracted from the database but only 134 were complete enough (or did not concern too many therapeutic lines) to keep them for analysis. Finally, 51 rationalizable ICSRs could be explained, which led us to propose a pharmacokinetic or pharmacodynamic interaction mechanism. Reports concerned more frequently women and half of the rationalizable ICSRs involved *Viscum album* and *Silybum marianum*. 5% of the ADRs described could have been avoided if clinicians had had access to the published information. It is also important to note that in 8% of the cases, the ADRs observed were life threatening. Phytovigilance should thus be considered more by health care professionals to best treat cancer patients and for better integrative care.

## Introduction

Phytovigilance^1^ concerns domains from pharmacovigilance to nutrivigilance. In Europe, phytovigilance is supported by the European Medicinal Agency (EMA) at pharmacovigilance level and by the European Food Safety Agency (EFSA) at nutrivigilance level. Globally, WHO promotes the clinical value and relevance of information on VigiBase^2,3^. This database has archived Adverse Drug Reactions (ADRs) of over 20 million Individual Case Safety Reports (ICSRs). Phytovigilance is particularly relevant for a patient’s real life during chronic treatments, such as cancer chemotherapy. Given the distress induced by diagnosis and treatment, there is a growing consensus towards considering cancer patients and their treatments more holistically. In general, western health professionals tend to discourage the use of phytotherapy due to the lack of relevant data, especially when combining an herb with Anti-Cancer Drug (ACD). In this article, we have focused on ADRs of patients undergoing an Anti-Cancer Drug (ACD) therapy together with the intake of one of 10 common herbs reported in VigiBase. We carried out a careful analysis to compare data on herb-drug interactions from the literature with real clinical situations. The societal goal of this project is to strengthen the knowledge of medical staff and to allow a more open exchange between patients and health care professionals.

## Methods

### Study design

A data extraction of ICSRs from the entire WHO database was performed by the Belgian Human Pharmacovigilance Evaluation cell on 2020-01-12. ICSRs containing at least one ACD and one of 10 representative herbs were extracted using ATC codes L01 antineoplastic agents or L02B hormone antagonists and related agents in a cancer clinic situation and herbs using their Latin binomial name. The herbs concerned are pineapple—*Ananas comosus* (L.) Merr., green tea—*Camelia sinensis (L.) Kuntze,* cannabis—*Cannabis sativa* L., black cohosh—*Cimicifuga racemosa (L.) Nutt.*, turmeric—*Curcuma longa L.*, echinacea—*Echinacea purpurea* (L.) Moench, St John’s wort—*Hypericum perforatum* L., milk thistle—*Silybum marianum (L.) Gaertn.* and ginger—*Zingiber officinale* Roscoe.

The choice of these herbs was made based on the current practice of phytotherapy in Europe to our knowledge. For *Curcuma longa,* a second VigiBase extraction was done with “curcumin” key word as the active ingredient. Duplicate ICSRs found were, thus, only mentioned once.

### Data curation

For each ICSR, the primary source country and reporter qualification were retrieved. The five categories of reporters’ qualifications were: physician, pharmacist, other health professional, consumer/non-health professional and unknown reporter qualification. Then a two-step data curation was carried out.

The first step aimed to select those with sufficient informative data available. Sufficient informative ICSRs include a minimum of at least one classified “suspected” or “interacting” anticancer drug with an herb and at least one ADRs. ICSRs containing too many therapeutic lines, conventional or not (> 5) were eliminated. In these cases, we are in polypharmacy (defined as regular use of at least five medications). Due to unspecific descriptions of the ADRs, and due the complexity of the pharmaceutical analyses in these cases, it seemed to us inappropriate to analyze these ICSRs. This is particularly the case with *Cannabis sativa*, which is often used to treat pain in palliative care situation concomitantly with many allopathic medications, or for *Zingiber officinale,* which is used in phyto-therapeutic complex formulas in traditional Asian medicine. ICSRs were not selected if only the term "drug interaction" was mentioned without indicating ADRs. Their main characteristics, i.e., suspected active ingredient, ADR (preferred term, in the Medical Dictionary for Regulation Activities—MedDRA) terminology, dechallenge, rechallenge and causality/seriousness of suspected and interacting drugs (when available) were gathered in Excel 2016 and we carried out an analysis of potential Herb-Drug Interactions (HDI).

For the second step, we worked on a rationalization of ADRs based on the literature. The potential pharmacokinetic (PK) and pharmacodynamic (PD) interactions of the suspected herbs and drugs were studied. For drugs, the Summary of Product Characteristics (SmPC) completed by Geneva University Hospital Cytochrome P450 tables^4^ or PubMed requests were used. For the herbs, monographs from EMA and from Stockley’s Herbal Medicines Interactions (2nd edition)^5^, reviews of clinical trials on clinicaltrial.gov, and a review of scientific publications using PubMed were consulted.

For each herb, a synthetic table was constructed indicating potential interactions between either OACDs (Oral ACDs) or PACDs (Parenteral ACDs) and the herbs, including the supposed natural secondary metabolites and mechanisms involved.

### Scoring

The final selection of rationalizable ICSR were scored at 2 levels according to Table 1. These scores are mentioned in the last two columns of Tables 2, 3, 4, 5 and 6.

**Table 1:** Scoring of clinical risk of HDI adapted from De Smet’s algorithm^6^ based on an alpha-numeric code.

Quality of evidence	Type of study
0	Pharmacodynamic (PD) animal studies; in vitro studies with a limited predictive value for the human in vivo situation; data on file
1	Incomplete published case reports (no re- or dechallenge, presence of other explanatory factors for the adverse reaction)
2	Well-documented, published case reports Retrospective analysis of case series
3	Controlled, published interaction studies in patients or healthy volunteers with surrogate endpoints
4	Controlled, published interaction studies in patients or healthy volunteers with clinically relevant endpoints

Category of HDI	Description (Examples)
A	No or insignificant clinical effect (Increased drug level without clinical symptoms)
B	Transient inconvenience (< 2 days) without residual symptoms (Fatigue, headache, nausea, amnesia)
C	Prolonged inconvenience (2–7 days) without residual symptoms
D	Failure of therapy for nonserious diseases prolonged (> 7 days) or permanent residual symptoms or invalidity (Toxic effects of ACD)
E	Increased risk of dying (Gastric hemorrhage, prolongation of QT interval, rhabdomyolysis)
F	Death

The first score concerns *causality*. The causality assessment found in the ICSRs were compared to literature review findings, and their concordance was rated using a gradation system with (*) or (**) where (**) is more robust than (*). (*) indicated that (i) there was a low degree of agreement between the causality assessment of the case (including when the ICSR was listed as "unlikely" in VigiBase) based on the literature or (ii) no causality assessment was found due to too many suspected interacting drug treatments or (iii) more than one route of administration was mentioned, which thus led to a complicated analysis of the interaction. (**) indicated that we agree with the causality assessment for at least one symptom. This causality score was indicated as “Concordance with ISCR conclusion” in Tables 2, 3, 4, 5 and 6.

The second score concerned *clinical risk* named “Level of Risk” in Tables 2, 3, 4, 5 and 6. We propose a comprehensive classification of risks based on an alpha-numeric gradation. The quality of the ADRs evidence was indicated by the numbers 0–4 and the seriousness of the potential ADRs by the letters A–F based on the classification system of De Smet^6^ (Table 1).

### Consent for publication

The Global ICSR database VigiBase was used as a data source for this article. Information in VigiBase comes from a variety of sources, and the probability that the suspected adverse effect is drug-related is not the same in all cases. Information in this article does not represent the opinion of the UMC or the World Health Organization.

## Results

**Figure 1:**
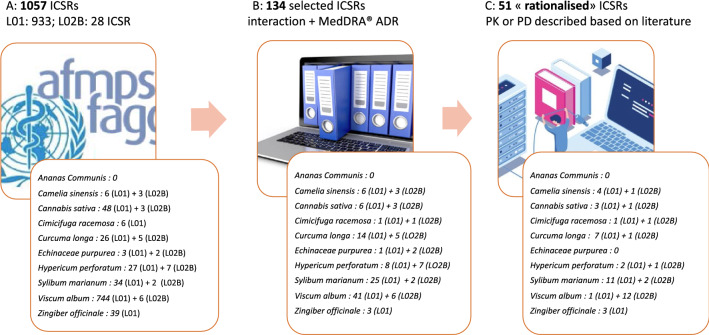
Flow chart from the selection of the 1057 ICSRs from VigiBase (**A**). In the first step, 1057 ICSR have been selected with 933 ICRS implicated drugs in L01 ATC class and 28 L02B drugs. At (**B**) step only 134 ICSR were selected for further investigation because they include at least “suspected” or “interacting” drugs/herb interaction and at least one adverse reaction. The last step (**C**) consists in the rationalization of the possible ADR due to PK or PD described interactions in literature.

Our analysis was based on ADRs reported in VigiBase when herbs were consumed at the same time as one or more drugs. Subsequently, 1057 ICSRs containing at least one ACD (Anti-Cancer Drugs in both ATC class L01 et L02B) and 1 of the 10 herbs chosen were extracted from the WHO database (Fig. 1).

**Figure 2:**
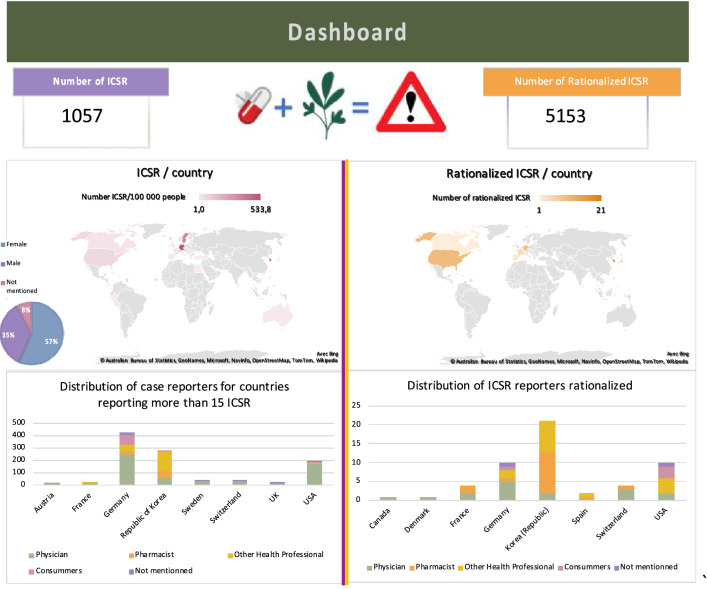
Dashboard with graphical representations of the geographical areas from which the declarations originate and the professional or consumer status of the declarants (draw with bing https://www.bing.com/).

A macroscopic examination of the data shows that physicians reported the majority of ICSRs (56%). Pharmacists reported 8% of them, other health professionals 22%, consumers/non-health professionals 10%, leaving 4% with unknown reporter qualification (Fig. 2).

The top three of countries reporting ADRs involving ACD and herbs (considering the number of inhabitants) are Germany, the Republic of Korea and the USA. There are more ICSR descriptions involving women (57%) than men (35%). Gender is not specified in 8% of ICSRs. Among retrieved ICSRs, cases involving *Viscum album* represented a substantial majority with 750/1057 ICSRs (71%). No ICSR was found for pineapple (Fig. 1), and no rationalized ICSR was possible on Echinacea.

The selection during the first step consisted of browsing the ICSRs manually to identify whether the description mentioned a suspected interaction or at least one adverse effect due to the association between the herb and the anticancer drug. After the first screening, only 134 ICSRs in VigiBase were complete enough to advance beyond the first step of selection. Noteworthy, around 600 ICSRs involved only *Viscum album* without any other medicine; 31/39 ICSRs involving *Zingiber officinale* were declared in Asia (either from the Republic of Korea or Japan in most cases) with more than 5 other herbs. In these cases, a relationship between one herb and the ACD is difficult to evaluate.

Only 51 ICSRs went on to the second step (Fig. 1). At this stage, the selection consisted of studying each ICSRs in detail and identifying whether an interaction mechanism could be identified based on the literature. In addition, the quality of the report does not seem to correlate with the professional status of the reporter (Fig. 2 and Table 1).

Among the remaining ICSRs, the predominant HDI was scored using two indicators, which are mentioned in the last columns of Tables 2, 3, 4, 5 and 6. Causability and clinical risk level were evaluated according to De Smet^6^. Causality assessments found in the ICSRs were compared to literature review findings; their concordance was rated using a gradation system. Clinical risk was evaluated considering (i) the quality of the evidence of the HDI considering peer reviewed publications; (ii) the seriousness of the resulting adverse reaction. In this article, a dichotomy was made between drugs given orally and parenterally (Tables 2, 3, 4, 5 and 6). The majority of the selected ICSRs concerned herb-OACDs interactions (29 ICSRs) and 22 ICSRs concerned PACDs. For all the herbs, tables described the rationalized interactions with the mechanism involved denoted in the central columns of said tables and the clinical adverse reaction observed.

### Green tea—*Camelia sinensis (L.) Kuntze*

Even if Cochrane review^7,8^ concluded that there is insufficient data to make recommendation on cancer incidence or cancer mortality, patients often consume green tea. This meta-analysis focuses on the cancer prevention impact based on prospective, controlled intervention studies and observational studies.

Pre-clinical data indicates that green tea exhibits possible interaction with PACD as bortezomib and OACD as tamoxifen^20,21^. In VigiBase, 5 cases were identified involving 4 drugs. In **CSa1, CSa2**, Table 2A, (−)-epigallocatechin gallate (EGCG) seems to be the key metabolite suspected of interacting with a drug known to be a Pgp substrate as erlotinib.

**Table 2:** (A) *Camellia sinensis* L. & (B) *Cannabis sativa* L.- ACD interactions among selected ICSRs.

A								
			Target	*Camellia sinensis (L.) Kuntze.*		Clinic		
DCI/ID/Indication	Level confidence‡†	Mechanism [ref]	Enz/transp/organ	Mechanism [ref]	Herb metabolites	Effect	Concordance with ISCR conclusion	Level of risk
**OACD Drug**								
Erlotinib/CSa1/Squamous cell carcinoma	SmPc	Subst^9^	Pgp	Inh/down-regulation of Pgp expression^10–13^	EGCG	PK: ↑ cutaneous rash	*	C-2
Erlotinib/CSa2/Non-small cell lung cancer	SmPc	Subst^9^	Pgp	Inh/down-regulation of Pgp expression^10–13^	EGCG	PK: ↑ dyspnea, hemoptysis	**	C-2
Imatinib/CSa3/Unknown	SmPc	Anemia^9^	Digestive iron absorption	↓ absorption of iron^14^	Catechins	PD: ↑ anemia	*	C-3
Anastrazole/Csa4/breast cancer recurrent	SmPc	Common hepatic side-effects	Liver	Hepatotoxicity^15^	EGCG	PD: ↑hepatocellular injury, cholestasis	*	D-2
**PACD Drug**								
Methotrexate/CSa5/Localized osteosarcoma	In vitro/in vivo	Subst^16–18^	OATP-A/B and AOX	Inh^19^	EGCG	PK: ↑ hepatotoxicity	*	C-2

B								
			Target	*Cannabis sativa L.Kuntze.*		Clinic		
DCI/ID/Indication	Level confidence‡†	Mechanism [ref]	Enz/transp/organ	Mechanism [ref]	Herb metabolites	Effect	Concordance with ISCR conclusion	Level of risk
**OACD Drug**								
Everolimus/CSb1/Unknown	SmPc	Subst^9^	CYP3A4; Pgp	CYP3A4 subst^26,35^/inh^27^ Pgp inh^29,35,36^	THC + metabolites 11-OH-THC and CBD	PK: ↑ nausea	**	B-3
Nintedanib/CSb2/Unknown	SmPc	Subst^9^	Pgp	Inh^29,35,36^	CBD	PK: ↑ Hepatic enzymes	**	C-0
Palbociclib/CSb3/Unknown	SmPc	Subst^9,37^	CYP3A4; Pgp	CYP3A4 subst^26,35^/inh^27^ Pgp inh^29,35,36^	THC + metabolites 11-OH-THC and CBD	PK: ↑ Tumour marker + Malaise	**	E-3
**PACD Drug**								
Carfilzomib/CSb4/Unknown	Case report	Dyspnea & cough^33,34^	CNS	respiratory distress syndrome^30–32^	n. k.	PD: ↑ chronic obstructive pulmonary disease	*	C-2

OACD: Oral Anti-cancer Drug; PACD: Parenteral Anti-cancer Drug; PD: pharmacodynamic; PK: pharmacokinetic; PgP:P-glycoprotein; CYP3A4: Cytochrom P450 isoform 3A4; CNS: Central Nervous System; Subst: Substrate; Inh: Inhibitor; n.k.: not known; EGCG: EpiGalloCatechin Gallate; AOX: Aldehyde Oxydase; OATP: Organic Anion Transporting Protein; CBD: Cannabidiol; THC: D9-Tetrahydrocannabinol; SmPc: Summary of product Characteristics. "Scoring" for the significance of the indicators "*", "**".

In **CSa2**, the patient was treated with a commercial product named Polyphenon E, a food supplement standardized^22,23^ in EGCG—200 mg a day. In vitro and animal studies^10,11,13^ describe increased blood levels of Pgp substrate in the presence of pure EGCG, a Pgp inhibitor, at concentrations from 1 µM whereas human blood concentrations after green tea ingestion can reach 1 mM^10^. A well-documented case study also mentioned increased blood levels of tacrolimus, a Pgp substrate, after green tea ingestion^12^, thus supporting previous descriptions. To our knowledge, no clinical trial was performed to assess this HDI. In **CSa3**, green tea is described as being involved in the decrease in iron absorption, while anemia is a very common adverse effect of imatinib. Several clinical studies on green tea have shown a noticeable decrease of 37% (and up to 99%) in iron absorption among healthy volunteers or patients. This mechanism is explained by the complexation of non-heme iron by the phenolic compounds of green tea, including catechins. Ahmad Fuzi et al*.* showed that a delay between non-iron heme and tea intake could reduce this interaction^14^. Two clinical trials studied iron absorption in women drinking different kinds of teas, and both led to the same conclusion^14,24^. In the only case (**CSa5**) involving a PACD, methotrexate (MTX), the interaction could be explained by inhibition of the organic anion transporting polypeptide (OATP)1A2. Indeed, EGCG has been described as an inhibitor of OATP1A2-mediated substrate transport on healthy volunteers^19^, while MTX is a substrate of this transporter in animal models^16^. In **CSa4**, a supplement containing a green tea extract (named Mega Green Tea Extract—725 mg a day containing 45% EGCG) could have worsened the hepatotoxicity of anastrazole. Although the mechanism of green tea hepatotoxicity remains unclear, a major safety concern exists when green tea is associated with other hepatotoxic compounds, thus enhancing the risk^15^.

### Cannabis—*Cannabis sativa L.*

For health care professionals, it is not appropriate to recommend cannabis for therapeutic use, even if the legislation concerning cannabis products is evolving. Cannabinoids are well known for their analgesic activities^25^. In **Csb1-3 **(Table 2B), we interpreted those cannabinoids as being the metabolites involved in the interaction. The concomitant administration of cannabinoids from Sativex with other CYP3A4 inhibitors leads to their increased blood levels in healthy human volunteers, suggesting that they could be CYP3A4 substrates^26^. Otherwise, Cannabidiol is described to inhibit CYP3A4 in vitro with IC_50_ of 11.7 µM in Human Liver Microsomes^27^ and Pgp from 5 µM on Caco-2 cells^28^. In vitro and animal studies have confirmed these pharmacokinetic characteristics of cannabinoids^29^, which could lead to increased blood levels of Pgp substrates, such as everolimus, nintendanib and palbociclib as described in **Csb1** to **Csb3** and to increased occurrence of adverse effects. Long-term Marijuana use is also known to cause CNS impairment^5^. **Csb4** describes additive effects (dyspnea and cough) that could have been aggravated by cannabis^30–32^. In the literature, to our knowledge, only two case reports mention a fatal acute respiratory distress syndrome with calfilzomib^33,34^. If the literature seemed to indicate a relevance of the PD interaction between cannabis and calfilzomib; however, a dechallenge of carfilzomib in **Csb4** was done without rapid recovery of the patient. This made uncertain the causality relationship between both products.

### Black cohosh—*Cimicifuga racemosa (L.) Nutt.*

Although studies are inconsistent, some clinical evidence of estrogenic activity support the use of black cohosh to treat climacteric symptoms including hot flushes, sweating, sleep disorders and nervous irritability^38^. Despite alternative findings in publications concerning patient follow-ups^39^ as well as meta-analysis of randomized, double-blind, and controlled clinical trials^40^, a very recent case of hepatotoxicity in patients consuming black cohosh has been published^41^. Effectively in its latest assessment report ^42^, EMA mentioned black cohosh as a potentially hepatotoxic, based on European pharmacovigilance signals. For this reason, we decided to extract ICSRs from VigiBase including “*Cimicifuga racemosa”* and “hepatic disorders” (Standardized MedDRA Queries). 160 reports (data not shown) were found. This high number of ICSRs supported the EMA report. In **CR1**,**2** (Table 3A), hepatotoxicity seemed to be due to OACDs and black cohosh additive adverse effects. Tsukamoto et al*.*^43^ showed that the triterpenes glycosides of black cohosh had a weak inhibitory effect on CYP3A4 while fingolimob (in **CR1**) is a substrate. This PK interaction could have increased the patient’s hepatotoxicity even if a meta-analysis demonstrated no evidence for hepatotoxicity^40^. Interestingly, in our selected cases, the same supplement was involved (Cimifemin—6.5 mg of dry extract—Ze 450), in case **CR1**, **2.** This product was used in retrospective observational studies^44,45^ without particular adverse effects. As both reports took place in Switzerland at close dates in 2016/17, it can be reasonably assumed that a particular batch had possibly been incriminated. Unfortunately, herbal food supplements do not have the same regulatory obligation in terms of quality as phytomedicine.

**Table 3:** (A) *Cimicifuga racemosa* L. & (B) *Curcuma longa* L.—ACD interactions among selected ICSRs.

A								
Drug			Target	*Cimicifuga racemosa (L.) Nutt.*		Clinic		
DCI/ID/Indication	Level confidence‡†	Mechanism [ref]	Enz/transp/organ	Mechanism [ref]	Herb metabolites	Effect	Concordance with ISCR conclusion	Level of risk
**OACD Drug**								
Fingolimob/CR1/Unknown	SmPC	Subst (+ Ind)^9^	CYP3A4 & Liver	weak inh^43^ & hepatotoxicity^42^	Triterpene glycosides (CYP3A4 inhibition)	PK/PD: ↑ hepatic damage, liver cholestasis, jaundice, epigastralgy, nausea, ↓ appetite	*	D-2
**PACD Drug**								
Trastuzumab+ Pertuzumab/CR2/Hepatic mestastasis	SmPC	Common hepatic side effect^9^	Liver	Hepatotoxicity^42^	*n.k.*	PD: ↑ hepatic damages @↓ appetite	*	D-2

B								
Drug			Target	*Curcuma longa L.*		Clinic		
DCI/ID/Indication	Level confidence‡†	Mechanism [ref]	Enz/transp/organ	Mechanism [ref]	Herb metabolites	Effect	Concordance with ISCR conclusion	Level of risk
**OACD Drug**								
Everolimus + Sunitinib/CL1/Pancreatic carcinoma	SmPC	Subst (Everolimus)^8^	CYP3A4/Pgp	Inh CYP3A4^39^/Pgp^40^	Curcumin	PK: ↑ Blood triglycerides increased, pain in jaw, dry skin	*	C-0
Ruxolitinib/CL2/Unknown	SmPC	Subst^8^	CYP3A4	Inh of CYP3A4^39^	Curcuminoids	PK: ↑ myalgia, fatigue, hemoglobin	*	C-0
Ibrutinib/CL3/Chronic lymphocytic leukemia	SmPC	Subst^8^	CYP3A4	Inh^39^	Curcuminoids	PK: ↑ thrombocytopenia, neutropenia	*	C-0
Ibrutinib/CL4/Chronic lymphocytic leukemia	SmPC	Subst^8^	CYP3A4	Inh^39^	Curcuminoids	PK: ↑ nausea, hypertension, hemorrhage, stomatitis, onychoclasis	*	C-0
Ibrutinib/CL5/Chronic lymphocytic leukemia	SmPC	Subst^8^	CYP3A4	Inh^39^	Curcumin	PK: ↑ dysgeusia, nausea, hypertension, hemorrhage, stomatitis, onychoclasis d	*	C-0
Methotrexate/CL6/Unknown	SmPC	Increased hepatic enzymes in blood^8^	Liver	Hepatotoxicity^41^	Curcuminoids	PD: ↑ hepatoxicity	**	D-2
Palbociclib/CL7/Breast carcinoma	SmPC	Subst^39^	CYP3A4	Inh^39^	Curcuminoids	PK: ↑ Hematotoxicity	*	C-0
** PACD Drug**								
Bortezomib/CL8/Pasma cell myeloma	HUG	Subst^4^	CYP3A4	Inh^47^	Curcuminoids	PK: ↑ Constipation@Red blood cell count decreased@Night sweats@Neuropathy peripheral@Rash macular	*	C-2

OACD: Oral Anti-Cancer Drug; PACD: Parenteral Anti-Cancer Drug; PK: pharmacokinetic; Pgp: P-glycoprotein; CYP3A4: Cytochrome P 450 isoform 3A4; CNS: Central Nervous System, Subs: Substrate; Inh: Inhibitor; n.k.: not known; Enz: enzyme; Transp: Transporter; ‡SmPC: Summary of product Characteristics; †HUG: University Hospital of Geneva^4^. "Scoring" for the significance of the indicators "*", "**".

### Turmeric—*Curcuma longa L.*

Turmeric is mainly used to treat digestive disorders, but it has many more uses in traditional Chinese medicine and Ayurveda. Its active compounds are the curcuminoids (3–5%); however, products vary considerably in their chemical composition^46^. Despite thousands of studies, robust scientific evidence on the effectiveness of turmeric in humans is lacking. Due to the low bioavailability of curcuminoids, doses needed to get an inhibition of hepatic CYP3A4 are usually not reached, but curcuminoids could inhibit intestinal CYP3A4^47^ and thus interact with OACD CYP3A4 substrates ^4^. It is the case in **CL1-5** (Table 3B) and **CL7-8**. Appiah-Opong et al*.* showed that curcumin inhibits CYP3A4 in human recombinant microsome preparations (IC_50_ 16.3 µM)^48^. Curcuminoids also inhibit Pgp (IC_50_ between 50 to 100 µM)^47^. In 2019, the British Committee on Toxicity of Chemicals in Food underscored the potential hepatotoxicity of curcumin on basis of in vitro and in vivo studies and case reports^49^.

In **CL6**, hepatotoxicity could be due to a PD interaction of MTX and turmeric. In this case, a 39-year-old woman was also consuming linseed oil, but no elements were found in the literature indicating hepatoxicity for this product.

### St John’s wort—*Hypericum perforatum L.*

Considerable clinical data, including Cochrane reviews^50–56^, have shown that St John’s wort is superior to placebo and is as effective as synthetic antidepressants in treating certain types of depression. Nonetheless, there is a high potential of interactions with other medicines. St John’s wort is a strong CYP3A4 inducer via one of its constituents, hyperforin^4^. In **HP1, 2** (Table 4), OACDs are CYP3A4 substrates, and this leads to PK interactions and thus a loss of any chance of recovery.

In **HP1**, the risk may have been greater because of the brief induction power of hyperforin on Pgp^4^. Hypericin, the other major constituent of St John’s wort can induce photosensitivity after UV exposure and generation of reactive oxygen species^57^. Hypericin and temozolomide^9^ share this adverse effect, which can explain the radiation-induced optic neuropathy observed in case report associated with **HP3**^58^.

**Table 4:** *Hypericum perforatum* L*.*- ACD interactions among selected ICSRs.

Drug			Target	*Hypericum perforatum *L.		Clinic		
DCI/ID/Indication	Level confidence‡†	Mechanism [ref]	Enz/transp/organ	Mechanism [ref]	Herb metabolites	Effect	Concordance with ISCR conclusion	Level of risk
**OACD Drug**								
Everolimus/HP1 /Myelodysplastic syndrome	SmPC	Subst^9^	CYP3A4/Pgp	CYP3A4 ind & Pgp inh^4^	Hyperforin	PK: ↓ drug blood level	**	E-4
Nilotinib/HP2/ hronic myeloid leukemia	SmPC	Subst^9^	CYP3A4	CYP3A4 ind^4^	Hyperforin	PK: ↓ drug blood level	**	E-4
**PACD Drug**								
Temozolomide/HP3/Unknown	SmPC	Photosensitivity^58^	cutaneous	Photosensitvity^58^	Hypericin	PD: Radiation induced optic neuropathy	**	D-3

OACD: Oral Anti-Cancer Drug; PACD: Parenteral Anti-Cancer Drug; PD: pharmacodynamic; PK: pharmacokinetic; Pgp: P-glycoprotein; CYP3A4: Cytochrome P 450 isoform3A4; Subst: Substrate; Inh: Inhibitor; Ind: Inducer; n.k.: not known; Enz: enzyme; Transp: Transporter; ‡SmPC: Summary of product Characteristics. "Scoring" for the significance of the indicators "*", "**".

### Milk thistle—*Silybum marianum (L.) Gaertn.*

Traditionally milk thistle is used to relieve the symptoms associated with the overindulgence of food and drink, including indigestion. Data to support its use to treat liver disease are mixed^59^. In vitro and animal studies have shown that silymarin or a mixture of milk thistle flavolignans, inhibits CYP3A4 and Pgp^60,61^. In animal model, CYP3A4 was also significantly downregulated compared to the control group with big amounts of silybin^62^.

Interactions in **SM1-7 and SM13** (Table 5) probably involve the CYP3A4.

**Table 5:** *Silybum marianum* L.- ACD interactions among selected ICSRs.

Drug			Target	*Silybum marianum (*L.) Gaertn*.*		Clinic		
DCI/ID/Indication	Level confidence‡†	Mechanism [ref]	Enz/transp/organ	Mechanism [ref]	Herb metabolites	Effect	Concordance with ISCR conclusion	Level of risk
**OACD Drug**								
Gefitinib/SM1/Unknown	SmPC	Subst^9^	CYP3A4	Inh^60,61^ downregulate ^62^	Silymarin	PK: ↑ pruritus	**	C-4
Gefitinib/SM2/Unknown	SmPC	Subst^9^	CYP3A4	Inh^60,61^ downregulate ^62^	Silymarin	PK: ↑ mouth dryness	*	B-4
Gefitinib/SM3/Unknown	SmPC	Subst^9^	CYP3A4	Inh^60,61^ downregulate ^62^	Silymarin	PK: ↑ somnolence	*	B-4
Gefitinib/SM4/Unknown	SmPC	Subst^9^	CYP3A4	Inh^60,61^ downregulate ^62^	Silymarin	PK: ↑ nausea, cutaneous cracks	*	B-4
Gefitinib/SM5/Unknown	SmPC	Subst^9^	CYP3A4	Inh^60,61^ downregulate ^62^	Silymarin	PK: ↑ prurit	*	B-4
Sorafenib/SM6/Unknown	SmPC	Subst^9^	CYP3A4	Inh^60,61^ downregulate ^62^	Silymarin	PK: ↑ diarrhea	**	B-4
Sorafenib/SM7/Unknown	SmPC	Subst^9^	CYP3A4	Inh^60,61^ downregulate ^62^	Silymarin	PK: ↑ alopecia	*	D-4
Sorafenib/SM8/Unknown	*In vitro*	Subst^68^	OATP 1B1/3	Inh^63,64^	Silymarin	PK: ↑nail discoloration	**	D-2
Imatinib/SM9/Leukemia	HUG	Subst^4^	CYP2C9	Inh^66,67^	Silymarin/silibinin	PK: ↑ anemia, pyrexia	*	D-4
Capecitabine/SM10/Unknown	HUG	Subst^4^	CYP2C9	Inh^66,67^	Silymarin/silibinin	PK: ↑ pruritus	**	B-4
Capecitabine/SM11/Unknown	HUG	Subst^4^	CYP2C9	Inh^66,67^	Silymarin/silibinin	PK: ↑ nausea	**	B-4
**PACD Drug**								
Methotrexate + Vincristine/ SM12/Unknown	*In vitro/in vivo*	Vincr: CYP3A4 & Pgp subst^4^; MTX: OATP subst^63^	CYP3A4/OATP-B1	Inh CYP3A4^60,61^ downregulate^62^ Inh OATP^69^	Silymarin/silibinin	PK: ↑ abdominal pain	*	B-2
Doxorubicine/SM13/Diffuse large B-cell lymphoma	SmPC	Subst^9^	CYP3A4	Inh^60,61^ downregulate^62^	Silymarin	PK: ↑ arrhythmia	*	C-4

OACD: Oral Anti-Cancer Drug; PACD: Parenteral Anti-Cancer Drug; PD: pharmacodynamic; PK: pharmacokinetic; Pgp: P-glycoprotein; CYP3A4: Cytochrome P 450 isoform3A4; OATP: Organic Anion Transporting Protein; Vinc: Vincristin; MTX: Methotrexate; Subst: Substrate; Inh: Inhibitor; Ind: Inducer; n.k.: not known; Enz: enzyme; Transp: Transporter; ‡SmPC: Summary of product Characteristics; †HUG: University Hospital of Geneva^4^. "Scoring" for the significance of the indicators "*", "**".

For **SM8,** potential interactions between silymarin (including silibin) and ACDs on OATP-B1, a liver specific uptake transporter, might be concerned. Wang et al*.* showed in vitro inhibitory power of flavolignans on OATP-B1 at 50 µM on HeLa cells^63^ while Fried et al., in a randomized clinical trial, observed blood concentrations of 2.1 µM of silybin-A after administration of 3 capsules of Legalon 140 mg a day^64^. This suggests a likelihood of PK interactions in present ICSRs that might have contributed to increasing the rare cutaneous side effect of ACD^65^. In **SM12**, describing abdominal pain with co-administration of Legalon with Vincristine and MTX, PK interaction with CYP3A4, PgP and OATP-B1 could be incriminated. Potential interactions in **SM9-11** could be explained by the effects of OACDs and milk thistle compounds on CYP2C9. Silibin A and B have shown inhibitory properties on CYP2C9 with IC_50_ of 8.2 to 18 µM and on recombinant CYP2C9 with IC_50_ of 2.4 to 19 µM depending on the genotypes^66^. A recent case report supports this hypothesis^67^.

In the majority of ICSR (12/13), the phytomedicine Legalon is suspected to interact. Legalon is a formulation of silymarin containing 108.2 mg Silymarin standardized on silibinin^9^. The robust chemical quality helps healthcare providers to argue potentially pharmacokinetic interactions.

### Mistletoe—*Viscum album (L.)*

In central Europe, European mistletoe preparations are not only among the most common types of treatments used in integrative medicine but also have been among the most prescribed cancer treatments in Germany in 2010. The dense literature on medical uses of mistletoe often gives indications that it improves the patient’s quality of life, but this is not considered conclusive yet^70,71^. While it may seem paradoxical that cytotoxic metabolites^72^ from mistletoe (as they kill cancer cells in vitro, down-regulate genes involved in tumor progression, malignancy, and cell migration and invasion) simultaneously helps the patients' well-being, some argue that Mistletoe increases the immune activity^73–75^. The second point that raises questions about these therapeutics comes from the specific products used. As demonstrated by our group, mistletoe extracts have different chemical compositions depending on the brand name and the host trees. This could be related to the manufacturing process using fermentation or not; Abnoba viscum is unfermented and the others are fermented^75–77^. 8 ICSRs are linked to Abnoba viscum products, 3 Helixor, 1 Iscador and 1 unknown (Table 6A). A multicentric observational study from Steele et al*.*^77^ in Germany shows that it is difficult to draw strong conclusions due to large variations in exposure frequencies of different preparation types. In our study, **VA1-6** detailed hematological toxicity mostly due to concomitant use of Abnoba products, **VA7-9** involved gastro-intestinal, **VA10-13** cutaneous disorders and **VA11-12** general disorders. Mistletoe extracts are to be considered in an original way, all cases might involve PD mechanisms and all implicated PACD except in **VA13** which concerns anastrazole. Data involving cytochromes and modification of metabolization^78–82^ were scarce but often reassuring.

**Table 6:** (A) *Viscum album* L. & (B) *Zingiber officinale* Roscoe—ACD interactions among selected ICSRs.

DCI/ID/Indication	Level confidence‡†	Mechanism [ref]	Enz/transp/organ	Mechanism [ref]	Herb metabolites	Effect	Concordance with ISCR conclusion	Level of risk
**A - OACD Drug**			**Target**	***Viscum album L.***		**Clinic**		
Anastrazole/ VA13/Unknown	SmPC	Rash^9^	cutaneous	Rash ^77^	Helixor M	PD: ↑ urticaria	*	B-3
**A - PACD Drug**			**Target**	**Herb**		**Clinic**		
Cisplatin/ VA1/Malignant neoplasm of cervix uteri	SmPC	Neutropenia^9^	Neutrophil	Neutropenia^78^	Abnovaviscum M 2 mg	PD: ↑ neutropenia	**	C-4
Oxaliplatin + fluorouracil/ VA2/Malignant neoplasm of bladder	SmPC	Neutropenia^9^	Neutrophil	Neutropenia^78^	Abnovaviscum M 0.02 mg	PD: ↑ febril neutropenia	**	C-4
Cisplatin + fluorouracil/ VA3/Malignant neoplasm	SmPC	Thrombopenia^9^	Thrombocyte	Thrombopenia^83^	Abnovaviscum F 20 mg	PD: ↑ thrombocytopenia	**	C-4
Carboplatin + paclitaxel/ VA4/Malignant neoplasm of ovari	SmPC	Leucopenia^9^	Leucocyte	Leucopenia^78^	Abnovaviscum M 2 mg	PD: ↑ leucopenia	**	C-4
Carboplatin + paclitaxel/ VA5/Malignant neoplasm	SmPC	Neutropenia^9^	Neutrophil	Neutropenia^78^	Abnovaviscum F 2mg	PD: ↑ neutropenia	**	C-4
Paclitaxel/ VA6/Malignant neoplasm	SmPC	Neutropenia^9^	Neutrophil	Neutropenia^78^	Abnovaviscum F 2mg	PD: ↑ neutropenia	**	C-4
Cisplatin + paclitaxel/ VA7/Malignant neoplasm of pyloric antrum	SmPC	Nausea^9^	Gastrointestinal disorders /cutaneous	Nausea^78^	Abnovaviscum M 20 mg	PD: ↑ nausea, rash, hot flush	**	B-3
Trastuzumab/ VA8/Breast cancer recurrent	SmPC	Nausea^9^	Gastrointestinal disorders	Nausea^78^	Iscador M	PD: ↑ nausea	**	B-3
Ifosfamide/ VA9/Malignant neoplasm of breast	SmPC	Nausea^9^	Gastrointestinal disorders	Nausea^78^	Abnovaviscum F 20 mg	PD: ↑ nausea	**	B-3
Topotecan/ VA10/Unknown	SmPC	Urticaria^9^	cutaneous	Urticaria^78^	Helixor A 100 mg	PD: ↑ urticaria	**	B-3
Cisplatin + fluorouracil/ VA11/Unknown	SmPC	Syncope^9^	Vascular/general disorders	Syncope^83^	Helixor A	PD: ↑ syncope	**	C-4
Epirubucine/ VA12/Breast cancer	SmPC	Fever^9^	General disorders	Pyrexia^83^	?	PD: ↑ fever	**	B-4
**B - OACD Drug**			**Target**	***Zingiber officinale Roscoe***		**Clinic**		
Imatinib/ZO1/Chronic myeloid leukemia	SmPC	Subst^9^	CYP3A4	Inh^85,86^	Gingerols	PK: ↑ hepatotoxicity	**	C-0
Dabrafenib/Trametinib/ZO2/Metastatic melanoma	SmPC	Subst^9^ (minor for Trametinib)	CYP3A4/Pgp	Inh^85,86^	Gingerols	PK: ↑ thrombocytopeniarectal hemorrhage	*	C-0
Crizotinib/ZO3/Adenocarcinoma of lung	SmPC	Subst^9^	CYP3A4 & Pgp	Inh^85,86^	Gingerols	PK: ↑ hepatic impairment	*	C-2

OACD: Oral Anti-cancer Drug; PACD: Parenteral Anti-cancer Drug ; PD: pharmacodynamic; PK: pharmacokinetic; PgP: P-glycoprotein; CYP3A4: Cytochrome P 450 isoform 3A4; CNS: Central Nervous System, Subs: Substrate; Inh: Inhibitor; n.k.: not known; SOC: System organ class; Enz: enzyme; Transp: Transporter; ‡SmPC: Summary of product Characteristics. "Scoring" for the significance of the indicators "*", "**".

### Ginger—*Zingiber officinale* Roscoe

Ginger is one of the most widely used herbal medicine and has a history of traditional use around the world. There is scientific evidence to support its use as antiemetic and for digestive complaints including chemotherapy-induced nausea and vomiting ^84,85^.

**In cases ZO1**,**2** and **ZO3** (Table 6B), potential interactions are certainly due to CYP3A4 for all of them and Pgp for **ZO2**,**3**. Tyrosine Kinase Inhibitors (TKI) involved in these cases are substrates of CYP3A4, Pgp or both. 6-gingerol is known to inhibit CYP3A4 and Pgp at blood concentrations from respectively 60 and 100 µM in vitro^85^, while 8-gingerol displays an IC_50_ of 8.7 µM on CYP3A4 in vitro^86^. Recent case reports seem to support these experimental data^87,88^ describing hepatic damages. Indeed, Bilgi et al*.* have published a cumulative hepatotoxicity with imatinib due to a PK interaction where ginseng inhibits CYP3A4^87^ while Revol et al*.* have demonstrated that crizotinib promotes severe hepatic cytolysis after the combination of ginger intake with this drug^88^. Again, the inhibition of CYP3A4 and Pgp was pointed.

## Discussion

Pharmacovigilance is a critical component of facilitating a clinician’s decision to alter or discontinue a patient’s therapy, including natural therapies. However, the increase in self-administration of OACD, requiring fewer clinical visits than PACD, may potentially lead to under-reporting of ADRs. Under-reporting is a setback in the early detection and assessment of safety problems. Significantly, only 1057 ICSR involving one of the ten most common herbs and one or more drugs from L01 and/or L02B ATC class were retrieved. Only 15 countries have reported more than 15 ICSRs. Our analysis is qualitative, not quantitative. We have chosen 10 plants based on our knowledge of consumption in Europe. From ICSRs reviewed, we sought to rationalize them consulting literature from clinical studies to in vitro data. The first factor that led us to reject the ICSR for interpretation was the presence of too many drugs or herbs (≥ 3). This was particularly the case for herbs involved in traditional Asiatic medicine. The second factor for excluding an ICSR was because it was not detailed enough, or the ADR was not clear. ICSRs were also excluded when the quality of the declaration was not sufficient for our interpretation (NB: the quality of the ADR description is not linked to the professional status of the declarants), thus leading finally to 51 ICSRs. It is noteworthy to mention that process of exclusion leads probably to underestimation of the number of herb-drug interactions but was necessary to ascertain the causality of the interaction.

The major risks associated with the use of herbal products and ACD are HDI. It is particularly undesirable in cancer management because of the narrow dose–effect relationship and toxicity of chemotherapeutic agents. Different ADRs have been observed in VigiBase, but the most common ones are liver or hematological toxicities and nausea. A particular interest was given to OACD for 2 main reasons. First, from a global/public health approach, OACD development is responsible for increasing health cost expenditures^89^. The economic sustainability of this care should not be thwarted by inappropriate complementary therapeutic habits. Secondly, in a more patient-centered approach, OACD not only implies greater autonomy and responsibility for their own care, but also raises adherence challenges^90^. However, patients are often not sufficiently educated about the potential risks of the simultaneous uses of different medications^91^. In these circumstances, the herb-OACD interaction risk is mathematically greater including (a) ADRs (due to an increase of AUC) and (b) risk for recurrence and mortality with no ADR observed but a decrease in ACD plasmatic concentrations due to the interaction. Twenty-nine declarations concerned OACD (vs. 22 PACD), and 31 involved PK interaction (*vs*. 19 PD & 1 both). The notations concerning mistletoe are original and imply only PD interactions and a large majority of PACDs.

The most common mechanism of HDI is PK with the herbal-mediated inhibition and/or induction of drug-metabolizing enzymes and/or transport proteins leading to the alteration of the body concentration of the active drug. Most mechanistic research published has focused on in vitro experiments. Extrapolating in vitro findings to predict clinical relevance is not trivial.

On the contrary, only 11 clinically relevant herb-drug interaction studies have been published at this time^92^.

In our opinion, the main limitation of this article comes from the lack of knowledge about the herb’s galenic form (herb powder, aqueous extract, hydro-alcoholic, essential oil or other food supplements form). So, it is difficult to rationalize/interpret the molecule(s) involved in the interaction. In general, no indication of the posology or herb treatment duration is present. Therefore, among other things, we conscientiously estimated the interaction using a 2 score indexes. In our study based on “real-life” patient ADRs, we sought to rationalize the ICSRs observed according to the literature to score the causality (using 1 or 2 *) and the clinical risk of encountering interactions (using alpha-numeric quotation). With this quotation, the highest risk was observed in interactions between cannabis or St. John's wort and TKIs when drug levels in plasma were decreased (or the tumor marker was increased), thus leading to a bigger risk of death.

Nevertheless, 5% of the 1057 ICSRs (51 cases) declared in VigiBase are rationalizable (or 40% of the 134 selected ICSRs as interpretable) by careful analysis of the literature. Moreover, 20% of those ICSRs (51 cases) were related to ADRs with a duration over 7 days and in 8% of the cases, the life risk was engaged due to HDIs. The highest number of ICSRs was observed with Milk thistle (Table 5). 25 of the ICSRs (about 50%) described in our tables involve protein kinase inhibitors. The 2 most represented NCIs in Table 5 (Milk Thistle) are gefitinib and sorafenib. It is important to note that the typology of ADRs is completely different with injected mistletoe with pharmacodynamic mechanisms.

The health care community has a great need for appropriate phytovigilance for the use of herb supplements. The importance of phytovigilance in oncology must be highlighted to improve safety and to offer cancer patients an improved quality of life during such a critical period of their lives. Lastly, we were surprised by the low total number of ICSRs. We thus strongly encourage more strenuous and detailed reporting and declarations of adverse events even in the context of herb-drug interactions. Risk minimization measures would be needed. In this purpose, health professionals should be informed about risks of interactions to reduce the occurrence of HDI. Various research groups are working on the subject. The NCCIH from NIH provides herbal monographs to enable clinicians to make informed choices (https://www.nccih.nih.gov/health/herbsataglance). Others are publishing combining computational, experimental and clinical approaches to better manage the use of plants^93,94^.

We are also working in this direction in order to produce a database available online and always up to date using machine learning (with HEDRINE for Herb Drug Interaction databasE at www.hedrine.ulb.be).

## Data Availability

The data that support the findings of this study are available from F. Souard, but restrictions apply to the availability of these data, which were used under license for the current study, and so are not publicly available. Data are however available from the authors upon reasonable request and with permission of J. Hamdani.
